# Beneficial Effects of Newly Isolated *Akkermansia muciniphila* Strains from the Human Gut on Obesity and Metabolic Dysregulation

**DOI:** 10.3390/microorganisms8091413

**Published:** 2020-09-14

**Authors:** Meng Yang, Shambhunath Bose, Sookyoung Lim, JaeGu Seo, JooHyun Shin, Dokyung Lee, Won-Hyong Chung, Eun-Ji Song, Young-Do Nam, Hojun Kim

**Affiliations:** 1 Department of Rehabilitation Medicine of Korean Medicine, Dongguk University, 814 Siksa-dong, Ilsandong-gu, Goyang-si 10326, Korea; yimuziyan@naver.com (M.Y.); shambose@yahoo.com (S.B.); sklim1972@naver.com (S.L.); 2 R&D Center, Enterobiome Inc., 814 Siksa-dong, Ilsandong-gu, Goyang-si 10326, Korea; jgseo@enterobiome.com (J.S.); jhshin@enterobiome.com (J.S.); dklee@enterobiome.com (D.L.); 3 Research Group of Healthcare, Korea Food Research Institute, Wanju 55365, Korea; whchung@kfri.re.kr; 4 Research Group of Gut Microbiome, Korea Food Research Institute, Wanju-gun 55365, Korea; songej486@hanmail.net

**Keywords:** *Akkermansia muciniphila*, probiotics, anti-obesity, metabolic disorders, gut microbiota, mice

## Abstract

The identification of new probiotics with anti-obesity properties has attracted considerable interest. In the present study, the anti-obesity activities of *Akkermansia muciniphila* (*A. muciniphila*) strains isolated from human stool samples and their relationship with the gut microbiota were evaluated using a high fat-diet (HFD)-fed mice model. Three strains of *A. muciniphila* were chosen from 27 isolates selected based on their anti-lipogenic activity in 3T3-L1 cells. The anti-lipogenic, anti-adipogenic and anti-obesity properties of these three strains were evaluated further in HFD-induced obese mice. The animals were administered these strains six times per week for 12 weeks. The treatment improved the HFD-induced metabolic disorders in mice in terms of the prevention of body weight gain, caloric intake and reduction in the weights of the major adipose tissues and total fat. In addition, it improved glucose homeostasis and insulin sensitivity. These effects were also associated with the inhibition of low-grade intestinal inflammation and restoration of damaged gut integrity, prevention of liver steatosis and improvement of hepatic function. These results revealed a difference in the distribution pattern of the gut microbial communities between groups. Therefore, the gut microbial population modulation, at least in part, might contribute to the beneficial impact of the selected *A. muciniphila* strains against metabolic disorders.

## 1. Introduction

Metabolic dysregulation is a cluster of metabolic abnormalities, including hyperglycemia, hyperinsulinemia and hyperlipidemia; it is a key indicator of obesity-related diseases, such as fatty liver disease, insulin resistance and type 2 diabetes [1]. Obesity is a complex disease involving the excessive accumulation of body fat and is a major global health problem. According to the World Health Organization (WHO) and other reports, chronic obesity develops over a long time and may induce specific metabolic abnormalities, such as pro-inflammatory states, dyslipidemia, high blood pressure, insulin resistance, glucose intolerance, non-alcoholic fatty liver disease (NAFLD), type 2 diabetes and some types of cancers [2,3,4,5]. Therefore, the prevention and treatment of obesity have attracted considerable attention globally [6].

The human intestine contains trillions of microorganisms [7], referred to collectively as the gut microbiota. Accumulating evidence suggests a close association of gut microbial communities with the host health and diseases [8]. In particular, an imbalance in the gut microbial population (dysbiosis) is linked to with various clinical conditions, including obesity [9]. Several studies on animals suggest that the consumption of a high-fat diet (HFD) alters the gut microbial communities. Such an event may eventually manifest augmented intestinal permeability as a result of the gut barrier disintegration, leading ultimately to the development of metabolic endotoxemia, inflammation and metabolic disorders [10,11,12,13]. Therefore, controlling gut microbial composition may be a therapeutic strategy for the prevention and treatment of obesity.

Accumulating evidence indicates that probiotics and their fermented food products have beneficial impacts [14]. In particular, probiotics support the homeostasis of the host gut microbial communities, improve immune disorders and combat inflammatory bowel disease, type 2 diabetes and obesity [15,16,17,18]. It has been revealed that heat-killed bacteria retain key probiotic effects and their favorable properties have been observed in in vitro, animal models, as well as clinical trials. Therefore, the usage of these bacteria has more advantages over live probiotics mainly because of the safety profile [19]. *Akkermansia muciniphila* (*A. muciniphila*), a mucin-degrading bacteria located in the intestinal mucus layer [20], is considered a promising probiotic candidate [21]. Several lines of evidence indicate the beneficial impact of *A. muciniphila* in the prevention and amelioration of metabolic disorders, obesity and associated complications [22,23,24]. Recently, in a proof-of-concept exploratory study, it has been found that, compared to the placebo, pasteurized *A. muciniphila* significantly improved insulin sensitivity and reduced insulinemia and plasma total cholesterol in the overweight and obese human volunteers [25].

Both the in vitro and in vivo properties and effects of a particular probiotic species are strain-specific [26]. This is supported further by the fact that different bacterial strains within a species manifest different host immunologic reactions [27]. In particular, most studies on *A. muciniphila* involve its type strain-BAA 835, which is isolated from the human intestinal tract [20]. The present study examined the protective activities of three new strains of *A. muciniphila* (EB-AMDK 10, EB-AMDK 19 and EB-AMDK 27) isolated from fecal samples against the obesity and metabolic dysregulation using 3T3-L1 preadipocytes and HFD-fed mice as in vitro and in vivo models, respectively. Furthermore, the possible mechanism(s) underlying such beneficial effects of these bacterial strains were analyzed.

## 2. Materials and Methods

### 2.1. Isolation, Culture and Screening of Bacterial Strains

The *A. muciniphila* strains were isolated from the fecal samples of 29 subjects registered in the present clinical study (Table S1) according to the method reported by Derrien et al. [20]. The Public Institution Bioethics Committee under the Ministry of Health and Welfare, Korea, approved this study (Approval number: P01-201705-31-002). The bacterial isolates were cultured, as described previously [23], in a basal medium supplemented with the following: 0.7% (*v/v*) clarified, sterile rumen fluid; 16 g/L soy-peptone; 4 g/L threonine; and a mixture of glucose and N-acetylglucosamine (25 mM each) (Sigma-Aldrich, St. Louis, MO, USA). The cultures were incubated in a serum bottle sealed with a butyl-rubber stopper at 37 °C under anaerobic conditions maintained by a gaseous phase of 182 kPa (1∙8 atm) N_2_/CO_2_ (80: 20, *v*/*v*) mixture. The cultures were washed with sterile phosphate-buffered saline (PBS) followed by centrifugation at 12,000× *g* for 5 min at 4 °C. The collected bacteria were then subjected to pasteurization at 70 °C in sterile PBS for 30 min, and the bacterial strains were stored at −80 °C until needed. The selection of 27 bacterial strains to be used for further in vitro screening was performed in accordance with a previous study [28].

### 2.2. Determination of Genetic Diversity of A. muciniphila Strains

Eight *A. muciniphila* strains were used for phylogenetic study to evaluate the genetic diversity (Figure S1). Among them, EB-AMDK 10, EB-AMDK 19, and EB-AMDK 27 were the three isolated strains used for the present study. Four *A. muciniphila* strains, BAA 835, 139, JCM 30893, and YL44 were selected from the reported *A. muciniphila* strains. *Akkermansia glycaniphila* APytT was chosen as an outgroup. We conducted the phylogenetic study on two targets, one for the 16S rRNA gene and the other for multi-locus sequence typing (MLST). Three genes, *ppk1, hemW,* and *lnt* were selected for MLST. The sequences were aligned in the ClustalO [29] with the default option. The phylogenetic trees were constructed by the maximum likelihood method based on the Tamura-Nei model [30]. The trees were drawn using MEGA X [31].

### 2.3. Cell Culture and Treatment

3T3-L1 murine preadipocytes were procured from Korean Cell Line Bank (KCLB, Seoul National University Cancer Research Institute, Seoul, Korea). The cells were seeded in a 96 well plate at a density of 2 *×* 10^4^ cells/well in Dulbecco’s modified Eagle’s medium (DMEM, Welgene Inc., Gyeongsan, Korea) supplemented with 10% fetal bovine serum (FBS, Thermo Fisher Scientific, Waltham, MA, USA) and 1% penicillin and streptomycin (Thermo Fisher Scientific, Waltham, MA, USA)). The cells were grown for four days in an incubator at 37 °C under a humidified atmosphere containing 5% CO_2_. The preadipocytes were then induced for differentiation by treating them with DMEM supplemented with 10% FBS, 0.5 mM 3-isobutyl-1-methylxanthine (IBMX, Sigma-Aldrich, St. Louis, MO, USA), 1 µM dexamethasone (Sigma-Aldrich, St. Louis, MO, USA) and 1 µg/mL insulin (Sigma-Aldrich, St. Louis, MO, USA) for two days. The cells were then maintained in DMEM containing 10% FBS and 1 µg/mL insulin for eight days to allow the intracellular deposition of lipid droplets, with a change in medium every alternate day. During the differentiation period (days 5–10 of cell culture, as mentioned above), the cells were also kept under treatment with the pasteurized twenty-seven selected strains and one reference type strain (ATCC BAA 835, American Type Culture Collection, Manassas, VA, USA) of *A. muciniphila* at a density of 1 × 10^8^ CFU/well.

### 2.4. Measurement of Intracellular Lipid Content

At day 10 of culturing along with the bacterial treatment, the adipocytes were subjected to Oil Red O staining. The cells were washed twice with cold phosphate-buffered saline (PBS), pH 7.4 without disturbing the cells and fixed with a 4% paraformaldehyde solution for 1 h at room temperature. The cells were then washed gently with PBS twice and then with 60% isopropanol, after which they were stained with the Oil Red O solution (Sigma-Aldrich, St. Louis, MO, USA) in 60% isopropanol for 15 min. The cells were washed repeatedly with cold distilled water and then examined under an inverted microscope (Olympus, Tokyo, Japan). Stained intracellular lipid droplets were dissolved in 100% isopropanol for 10 min, and the resulting solution was transferred to a 96-well plate. The absorbance of the stain was read at 500 nm using a microplate reader (Molecular Devices, San Jose, CA, USA).

### 2.5. Cellular RNA Extraction and Quantitative Polymerase Chain Reaction (qPCR)

At the end of the experimental schedule, RNA was extracted from the adipocytes that had been treated with three strains of *A. muciniphila* selected from the 27 screened isolates, i.e., *A. muciniphila* 10 (EB-AMDK 10; Source: Male, Age 7, BMI 17.4), *A. muciniphila* 19 (EB-AMDK 19; Source: Female, Age 35, BMI 23.3), *A. muciniphila* 27 (EB-AMDK 27; Source: Female, Age 45, BMI 23.1) and *A. muciniphila* type strain (ATCC BAA 835). After washing gently with PBS, the cells were lysed and the total cellular RNA was extracted using a TRIzol^®^ reagent (Life Technologies, Carlsbad, CA, USA) according to the manufacturer’s instructions. Qualitative and quantitative analyses of the extracted RNA were performed by measuring the optical densities at 260 nm and 280 nm using a nanodrop spectrophotometer (Implen, Munich, Germany). To prepare the cDNA, 1 µg of RNA was reverse transcribed using an oligo-(dT) 18 primer and a cDNA RT PreMix kit (Bioneer, Daejeon, Korea) according to the manufacturer’s protocol. Real-time qPCR was carried out on a Light Cycler 480^TM^ platform (Roche, Mannheim, Germany) in a 96-well plate using an SYBR^®^ Green real-time PCR Master Mix kit (Toyobo, Tokyo, Japan). The amplification reactions were accomplished according to the instructions from the kit manufacturer in a 20 µL volume of PCR mixture, containing 1 µL of cDNA, 10 pmol of each reverse and forward primer of a given gene (Bioneer, Table S2), 10 µL of SYBR^®^ Green real-time PCR Master Mix and 8 µL of nuclease-free water. The obtained data were processed and analyzed using dedicated Light Cycler software (version 1.2, Roche Applied Science) and normalized using glyceraldehyde-3-phosphatase dehydrogenase (GAPDH) as a housekeeping gene. The relative gene expression was measured using the standard quantification of 2^−∆Ct^, where C_t_ refers to the crossing threshold value determined using the software with ∆C_t_ = (C_t-target gene_-C_t-GAPDH_).

### 2.6. Animals and Treatments

Male C57BL/6 mice, aged seven weeks (body weight 19 ± 2 g), were purchased from Daehan Biolink Co. Ltd. (Eumseong, Chungbuk, Korea). All mice were housed in a 12-h light/dark cycle under a constant temperature of 20 ± 3 °C, humidity of 55 ± 5% with access to autoclaved distilled water and a normal chow diet (Feedlab, Guri-si, Gueonggi-do, Korea) ad libitum over two weeks of acclimatization. The study was approved by the ‘Institutional Animal Care and Use Committee’ of the Dongguk University (IACUC-2019-009-01) and carried out in strict accordance with the recommendations of the “Guide for the Care and Use of Laboratory Animals” (Institute for Laboratory Animal Research, Committee for the Update of the Guide for the Care and Use of Laboratory Animals, National Research Council of The National Academies, USA; The National Academies Press: Washington, DC, USA, 2011). Both normal low-fat and high-fat diets were obtained from Research Diets, Inc. (New Brunswick, NJ, USA). The normal low-fat diet contained 19.2% protein, 67.3% carbohydrate and 4.3% fat (total calories 3.85 kcal/g, 10% calories from fat). The high-fat diet (HFD) contained 26.2% protein, 26.3% carbohydrate and 34.9% fat (total calories 5.24 kcal/g, 60% calories from fat).

After acclimatization, sixty-nine mice were divided randomly into seven groups as follows: Normal low-fat diet group (NOR, *n* = 12), high-fat diet group (HFD, *n* = 12), *Garcinia cambogia* -treated high-fat diet group (GC, *n* = 9), *A. muciniphila* type strain BAA 835-treated high-fat diet group (BAA 835, *n* = 9), *A. muciniphila* 10-treated high-fat diet group (EB-AMDK 10, *n* = 9), *A. muciniphila* 19-treated high-fat diet group (EB-AMDK 19, *n* = 9) and *A. muciniphila* 27-treated high-fat diet group (EB-AMDK 27, *n* = 9). The animals were given free access to the diet and sterile water throughout the treatment period. The body weights and food intakes of the mice were measured weekly and the calorie intake was estimated from the food consumption. The powder form of the *Garcinia Cambogia* extract (iBT international Bio-Tech, Gunpo-si, Gyeonggi-do, Korea) was dissolved in sterile PBS (pH 7.4) and administered to the mice in the GC group orally at a dose of 200 mg/kg body weight. The BAA 835, EB-AMDK 10, EB-AMDK 19 and EB-AMDK 27 groups were administered the respective pasteurized bacterial strains orally, as mentioned above, in sterile PBS orally at a dose of 1 × 10^8^ CFU/animal. The NOR and HFD groups were administered sterile PBS as a vehicle instead of GC or bacterial strains. The treatments with the above-mentioned regimens were performed six times per week. Fresh fecal samples were collected at week 0 and week 12 of the experimental schedule and stored at −80 °C for further microbiological analysis. At the end of the study period at week 12, the mice were sacrificed under anesthesia (Zoletil 50, tiletamine-zolazepam, Virbac S.A, Carros, France) following a 12 h fast. Blood was collected rapidly through a cardiac puncture, as described previously [32]. The liver, intestine and mesenteric adipose tissue were removed promptly, washed in ice-cold PBS, blotted and weighed. Some portions of the tissues, either untreated (for Western blotting) or treated with Invitrogen RNAlater stabilization solution (Thermo Fisher Scientific, Waltham, MA, USA, for RNA preparation), were immediately snap-frozen in liquid nitrogen before storing at −80 °C for further use. Other portions of the tissues dedicated to histological analysis were fixed in 4% formalin (Junsei, Tokyo, Japan) and stored at 4 °C until processed. The sera were separated after clotting and centrifuging the blood samples at 2000× *g* for 15 min at 4 °C and finally stored at −80 °C for further biochemical analyses.

### 2.7. Oral Glucose Tolerance Test (OGTT)

OGTT was performed at week 11 of the experimental schedule. Briefly, the 14h-fasting-adapted mice were administered a sterilized aqueous solution of glucose (Sigma-Aldrich) by oral gavage at a dose of 2 g/kg body weight. Blood was collected from the tail vein of the animals at five post-treatment periods (0, 30, 60, 90 and 120 min). Measurement of the glucose level in the serum samples was performed using glucose test strips and a handheld glucose meter (Accu-Chek Active; Roche Diagnostics, Rotkreuz, Switzerland) with a detection level ranging from 1.7 to 33.3 mmol/L. The glucose area under the curve (AUC) was estimated by plotting the glucose concentration (mmol/L) as a function of time (min).

### 2.8. Analyses of the Serum Biochemical Parameters

Colorimetric measurements of the serum concentrations of the total cholesterol (TC), triglyceride (TG), glutamic pyruvic transaminase (GPT) and glutamic oxaloacetic transaminase (GOT) were performed using commercial assay kits (Asan Pharmaceutical, Seoul, Korea). The serum insulin concentration was determined using an ELISA kit (Morinaga, Yokohama, Japan). The insulin resistance index (HOMA-IR) was calculated using the following formula: Fasting serum insulin (microU/L) × fasting serum glucose (mmol/L)/22.5.

### 2.9. Histological Studies

The formalin-fixed tissues were embedded in paraffin blocks and sectioned at a 4 μm thickness using a microtome (Leica RM2235, Leica, Nussloch, Germany). For hematoxylin and eosin (H&E) staining of the liver and mesenteric adipose tissues, the sections were placed on positively charged silicon-coated glass slides, deparaffinized with xylene and then rehydrated in a graded series of ethanol. The sections were finally stained with H&E (Sigma-Aldrich). The stained tissues were then dehydrated in a graded series of ethanol and mounted. For Alcian blue (AB) and Oil Red O staining of the intestine and liver, respectively, the same procedure for the tissue preparation described above for H&E staining was followed. The intestinal and hepatic tissue sections were then stained with AB using a commercial kit (Abcam, Cambridge, UK) and Oil Red O (Sigma-Aldrich), respectively. The stained tissue sections were examined under an inverted microscope (Olympus, Tokyo, Japan), and the images were acquired using a digital camera (Olympus, Tokyo, Japan). The liver steatosis area, as a typical measurement of the macrovesicular steatosis region and the Oil Red O-stained area, was determined from the images using dedicated image analysis software (Image-Pro Plus 6.0; Media Cybernetics, Inc., Rockville, MD, USA). The AB-positive area, villus length and the number of the goblet cells in the intestinal tissue sections were determined using ImageJ, which is a public domain Java-based image-processing program developed at the National Institutes of Health, Bethesda, MD, USA. For the AB-positive area, the measurements were taken using images of the entire colonic section per animal. The villus length was measured from the base to the top of the villus. Five to six villi were selected randomly from different fields in each colonic section to determine the mean villus length of each animal. Goblet cells were counted in all crypts present in the entire colonic section and three sections were chosen randomly for this measurement per animal.

### 2.10. Bacterial DNA Extraction and Sequencing

The bacterial genomic DNA was extracted from the fecal samples using a QIAamp stool DNA mini kit (QIAGEN, Hilden, Germany), as described previously [11]. Amplification of the V1-V2 region of 16S rRNA gene sequences was performed using a C1000 Touch thermal cycler with a 96-deep-well reaction module (Bio-Rad, Hercules, CA, USA). The primer set used for this reaction contained a unique 8-base barcode to tag the PCR products of the samples. The PCR products were pooled and the amplicons were purified using a QIAquick PCR purification kit (QIAGEN, Hilden, Germany). The sequencing reactions were carried out on an Ion Torrent PGM system (Thermo Scientific, DE, USA) according to the manufacturer’s instructions. The high quality reads for further bioinformatics analyses were selected, and all of the effective reads from the samples were clustered into operational taxonomic units (OTUs) based on a 97% sequence similarity (SILVA database: http://www.arb-silva.de). This was followed by the selection of the representative sequence using the Quantitative Insights into Microbial Ecology (QIIME) software package [33]. Briefly, the filtering of high-quality raw data was based on the removal of sequences that lacked the V1-V2 primers or barcode sequence, contained a short read length (<300 bp) or demonstrated low quality reads (average quality score < 20). The clustering pattern of the microbial composition in each experimental group was examined by UniFrac-based principal coordinated analysis (PCA) of the OTUs data to reveal the total structural changes in the gut microbial communities. To distinguish the taxa with varying abundances among the groups, a linear discriminant analysis effect size (LEfSe) assessment was carried out using a web-based program (Galaxy, available online: http://huttenhower.sph.harvard.edu/galaxy). For this study, the threshold of the logarithmic linear discriminant analysis (LDA) score was set to >2.0, and the alpha value of the factorial Kruskal–Wallis test among the classes was set to <0.05.

### 2.11. Tissue RNA Extraction and Quantitative Polymerase Chain Reaction (qPCR)

The total RNA was isolated from the liver, intestine and adipose tissues using a TRIsure^TM^ reagent (BIOLINE, Meridian Life Science, TN, USA) according to the manufacturer’s protocol. qPCR of the samples and subsequent data analyses were performed similarly, as described above for the 3T3-L1 cells. Table S2 lists the primers used.

### 2.12. Western Blotting

The liver and colon tissues were homogenized on ice in an extraction buffer containing RIPA buffer (Biosesang, Gyeongsangnam-do, Korea) supplemented with cOmplete^TM^ Mini protease inhibitor cocktail (Roche Diagnostics GmbH, Mannheim, Germany) and Xpert phosphatase inhibitor cocktail (GenDEPOT LLC, Houston, TX, USA). The tissue homogenates were centrifuged at 13,000× *g* for 15 min at 4 °C. The resulting supernatants were collected and stored at −80 °C until used. The protein concentrations of the supernatants were measured using a Bradford assay (Bio-Rad, Hercules, CA, USA) according to the manufacturer’s instructions. Thirty micrograms of protein from each sample were denatured by heating at 95 °C for 5 min in Laemmli sample buffer (Bio-Rad, Hercules, CA, USA) containing 5% β-mercaptoethanol. The protein was then separated on a 10% acrylamide-bisacrylamide gel by SDS-PAGE (Bio-Rad, Hercules, CA, USA) using a constant voltage of 100 V for 80 min and finally transferred to a 0.45 µm polyvinylidene fluoride (PVDF) membrane (Amersham^TM^, GE Healthcare, Munich, Germany) using a Mini Trans-Blot^®^ electrophoretic transfer cell device (Bio-Rad). The membranes were blocked by incubation with Tris-buffered saline containing 0.1% Tween 20 (TBST) and 5% skim milk (Becton Dickinson, Sparks, MD, USA) for 1 h at room temperature. After washing with TBST, the membranes were incubated overnight at 4 °C in TBST containing 5% bovine serum albumin (Life Science, Solon, OH, USA) with the following primary antibodies: Anti-PPARγ (Millipore, Burlington, MA, USA), anti-SREBP1 (Abcam, Cambridge, UK), anti-occludin (Santa Cruz, Baker, OR, USA) and anti-claudin-1 (Santa Cruz, Baker, OR, USA) antibodies. The membranes were then washed with TBST and incubated for 1h with horseradish peroxidase (HRP)-conjugated anti-mouse or anti-rabbit immunoglobulin G (IgG) secondary antibodies (Santa Cruz, Baker, OR, USA) depending on the origin of the primary antibodies. The anti-β-actin antibody (Santa Cruz, Baker, OR, USA) was used as a loading control to normalize the protein loading and transferring processes. The immunoreactive protein bands were visualized using a Bio-Rad ChemiDoc XRS imaging system (Bio-Rad, Hercules, CA, USA) with an ECL reagent (Neuronex, Seoul, Korea). The band intensities were analyzed using Image-Pro Plus 6.0 software.

### 2.13. Statistical Analyses

All the data are expressed as the arithmetic means ± standard error (SEM). GraphPad Prism 5 (GraphPad, San Diego, CA, USA) was used for statistical analyses, which were based on the two-sided unpaired Student’s *t*-test or one-way ANOVA with a Bonferroni test as a post-hoc test to correct for multiple comparisons unless indicated otherwise. *p* value < 0.05 was considered statistically significant.

## 3. Results

### 3.1. Screening of A. muciniphila Strains

**Figure 1:**
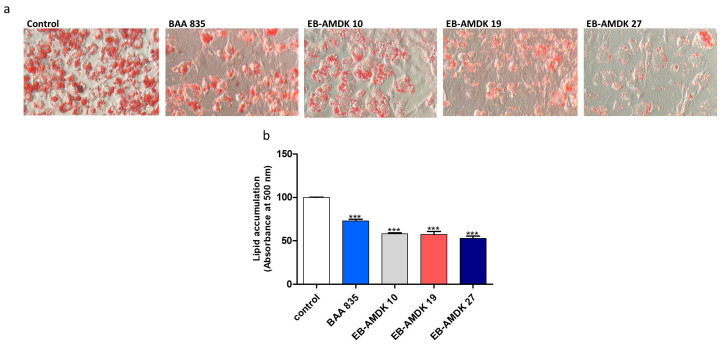
Effects of *Akkermansia muciniphila* strains on lipid accumulation in 3T3-L1 adipocytes. (**a**) After differentiation, the intracellular lipid droplets of adipocytes in different experimental groups were visualized under a microscope at 200× magnification upon staining of the cells with Oil Red O. (**b**) Intracellular deposition of the lipids was measured after extraction and reading the absorbance at 500 nm. The data are presented as the mean ± SEM. *** *p* < 0.001 versus the control group.

Twenty-seven of the laboratory-isolated pasteurized strains of A. muciniphila were screened based on their inhibitory effect on lipogenesis in differentiating adipocytes using the 3T3-L1 mouse embryonic fibroblast cell line as a model. For this study, the type strain (ATCC BAA 835) was used as a reference. Oil Red O staining showed that the treatment of 3T3-L1 cells with the A. muciniphila strains, including BAA 835, suppressed the intracellular lipid accumulation significantly in a dose-dependent manner. On the other hand, among the laboratory-isolated bacterial strains, EB-AMDK 10, EB-AMDK 19 and EB-AMDK 27, had the most potent anti-lipogenic activity (Figure 1a,b). Accordingly, these three strains were selected for further in vitro and in vivo assessments of their anti-lipogenic, anti-adipogenic and anti-obesity properties, as described below.

### 3.2. Impact of A. muciniphila Treatment on the Adipogenic and Lipogenic Gene Expressions in Adipocytes

Treatment of 3T3-L1 cells with the three pasteurized *A. muciniphila* strains selected significantly downregulated the gene expressions of adipogenic/lipogenic markers (PPARγ, C/EBPα and CD36) and lipogenic enzymes (aP2, ACC1, LPL and FAS) (Table 1). Interestingly, the mRNA levels of PPARγ, C/EBPα and CD36 were significantly lower in all three *A. muciniphila* strains-treated cells selected compared to the cells exposed to the *A. muciniphila* type strain BAA 835. Furthermore, the levels of LPL gene expression in the EB-AMDK 10 and EB-AMDK 19-treated cells were significantly lower than in the BAA 835-treated cells.

### 3.3. In Vivo Effects of A. muciniphila on the Vital Obesity Parameters in the HFD-Fed Mice

**Figure 2:**
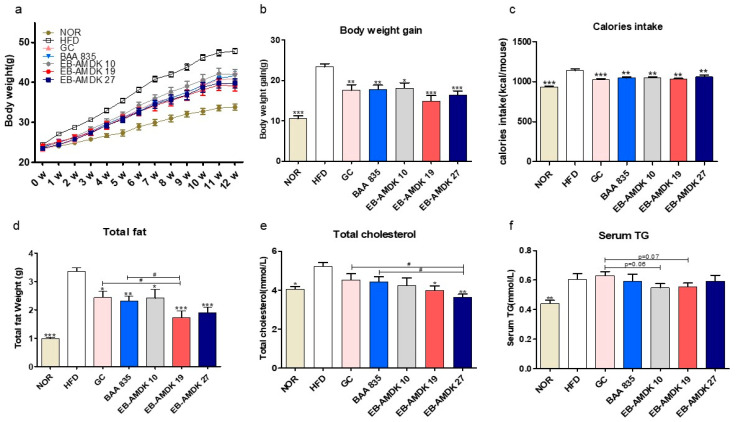
Effects of *A. muciniphila* treatment on the vital obesity parameters in high fat-diet (HFD)-fed mice. (**a**) Bodyweight measured weekly, (**b**) total body weight gain, (**c**) caloric intake, (**d**) weight of total fat (subcutaneous fat + epididymal fat + mesenteric fat), (**e**) serum level of total cholesterol (TC) and (**f**) serum triglyceride (TG) level in different experimental groups. The data are presented as the mean ± SEM (*n* = 9–12). Statistical analyses were performed by one-way ANOVA or a Student’s t-test. * *p* < 0.05, ** *p* < 0.01 and *** *p* < 0.001 versus the HFD group. ^#^ *p* < 0.05.

At the end of the experimental schedule in week 12 (Figure 2a), the body weight gain (Figure 2b); caloric intake (Figure 2c); energy efficiency, weights of subcutaneous fat, epididymal fat, mesenteric fat (Table S3) and total fat (Figure 2d); and serum TC (Figure 2e) and TG (Figure 2f) levels were significantly higher in the HFD group than in the normal group. On the other hand, the body weight gain, caloric intake, weights of the mesenteric fat and total fat were decreased significantly in the HFD-fed mice upon treatment with all three selected pasteurized *A. muciniphila* strains. In contrast, exposure of the HFD group to EB-AMDK 19 and EB-AMDK 27, but not to other treatments, reduced the energy efficiency, subcutaneous fat weight and serum TC concentration significantly. Moreover, the epididymal fat weight in the HFD-fed animals was reduced significantly by GC, EB-AMDK 19 and EB-AMDK 27. Furthermore, the liver weight (Table S3) was significantly lower in the EB-AMDK 27 group than in the BAA 835 group.

### 3.4. A. muciniphila Improved Insulin Sensitivity and Glucose Homeostasis in the HFD-Induced Mice

**Figure 3:**
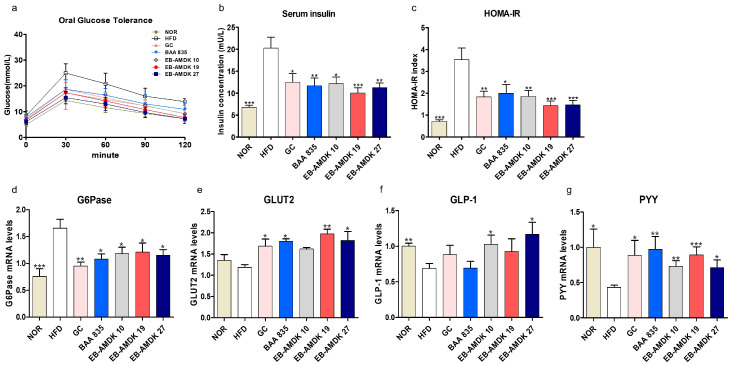
*A. muciniphila* strains improved insulin sensitivity and glucose homeostasis in HFD-induced obese mice. (**a**) Serum glucose levels at different time-points in oral glucose tolerance tests (OGTT). (**b**) Serum insulin level and (**c**) HOMA-IR. mRNA levels of glucose homeostasis (**d**) G6Pase and (**e**) GLUT2 in the hepatic of each group (*n* = 9–12 mice/group). mRNA expression of related markers of gut hormones (**f**) GLP-1 and (**g**) PYY were measured in the intestinal tissue (*n* = 9–12 mice/group). The data are represented as the mean ± SEM. Statistics were performed with one-way ANOVA or Student’s t-test. * *p* < 0.05, ** *p* < 0.01 and *** *p* < 0.001 versus the HFD group.

The in vivo impact of pasteurized *A. muciniphila* treatment on insulin sensitivity and glucose homeostasis in mice fed a high-fat diet was next determined. The results showed that the fasting serum glucose level in the samples collected at 0 min of OGTT (immediately before glucose ingestion) was significantly higher in the HFD group than the normal group (Figure 3a and Figure S2a). Treatment of the HFD-fed mice with EB-AMDK 19 and EB-AMDK 27, but not other regimens, depleted this parameter significantly. In OGTT, for both the normal and HFD groups, the time to reach the peak serum glucose concentration (peak OGTT glucose level) occurred at 30 min; the latter group exhibited significantly higher values than the former (Figure 3a and Figure S2b). In this period, however, the serum glucose levels in all the three selected *A. muciniphila* groups was significantly lower than the HFD group. The OGTT AUC (Figure S2c), serum insulin level (Figure 3b), HOMA-IR (Figure 3c) and hepatic gene expression of G6Pase (Figure 3d), an enzyme involved in glucose production, were significantly higher in the HFD group than in the normal group. Nevertheless, treatment of the HFD group with the three selected *A. muciniphila* strains reduced the levels of these four parameters significantly. Furthermore, OGTT AUC was significantly lower in the EB-AMDK 19 group vs. the BAA 835 group. In contrast, this parameter in the EB-AMDK 27 group was significantly lower than both the GC and BAA 835 groups. These results also showed that the hepatic gene expression of the facilitative glucose transporter GLUT2 was increased significantly by exposure to EB-AMDK 19 and EB-AMDK 27 (Figure 3e). This study examined further the impacts of *A. muciniphila* in the HFD-fed animals on the colonic gene expression of GLP-1 and PYY (Figure 3f,g), which are the gut hormones with appetite suppressing and anti-diabetic plus anti-obesity properties, respectively. The gene expression of both of these hormones was lower in the HFD group in response to feeding HFD. On the other hand, treatment of the HFD-fed animals with all the *A. muciniphila* strains and GC elevated the mRNA level of PYY significantly. Exposing the HFD group to EB-AMDK 10 and EB-AMDK 27, but not other treatments, upregulated the expression of GLP-1 gene significantly.

### 3.5. Prevention of HFD-Induced Hepatic Lipid Accumulation and Liver Damage by A. muciniphila

The normal and HFD groups showed similar serum GOT levels (Figure S3a). On the other hand, treatment of the HFD-fed mice with EB-AMDK 10 and EB-AMDK 19 reduced this parameter significantly. The serum GPT level in the HFD group was significantly higher than the normal group, indicating an HFD-induced liver injury Exposure of the HFD group to all *A. muciniphila* strains caused a significant decrease in the serum level of this enzyme (Figure S3b).

**Figure 4:**
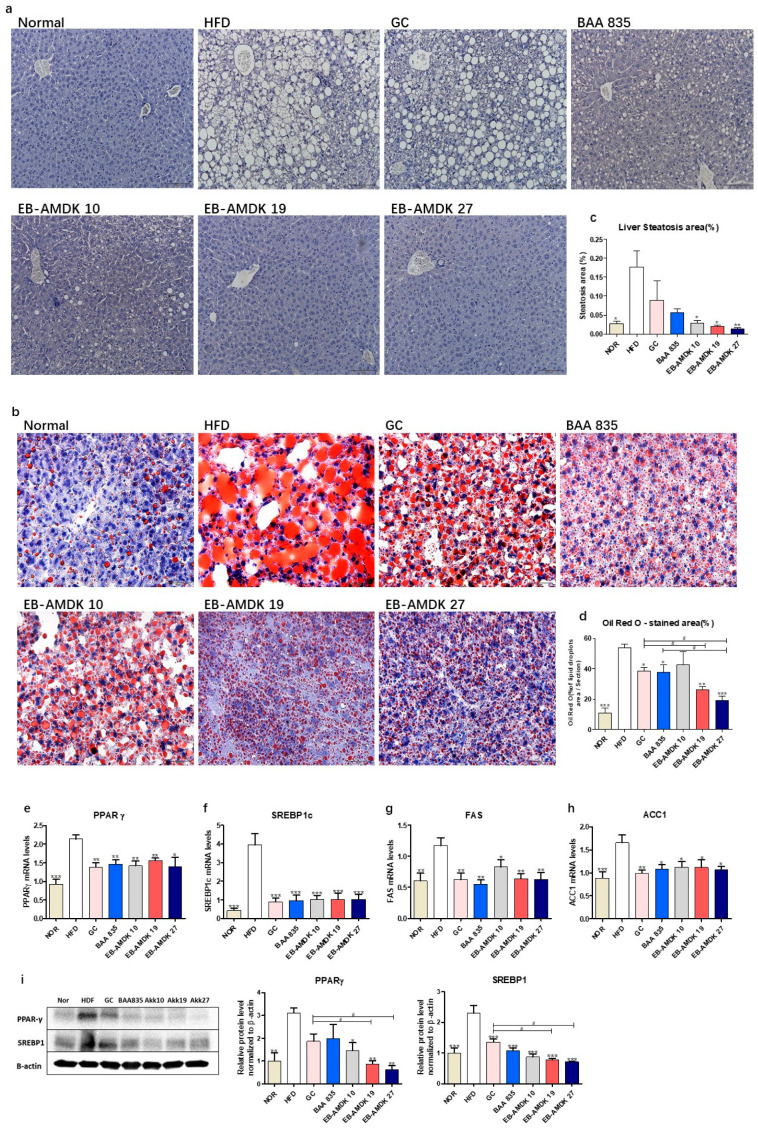
*A. muciniphila* administration alleviated hepatic steatosis and improved hepatic function in mice. (**a**) Histological analysis of H&E stain on the liver sections of mice (*n* = 3 per condition, scale bar, 100 μm). (**b**) Representative Oil Red O staining for fat deposition measurement in the liver. (**c**) The liver steatosis area. Liver steatosis characterized by micro- and macrovacuolization. (**d**) Oil Red O stained fat deposition area. (*n* = 3 per condition, scale bar, 50 μm). Representative mRNA expression of (**e**) PPARγ, (**f**) SREBP1c, (**g**) FAS and (**h**) ACC1 in the liver tissue from mice (*n* = 9–12 mice/group). (**i**) The protein expression of the liver tissue (*n* = 4 mice/group). The data are represented as the mean ± SEM. Statistics were performed with one-way ANOVA or a Student’s t-test. * *p* < 0.05, ** *p* < 0.01 and *** *p* < 0.001 versus the HFD group. ^#^ *p* < 0.05.

As expected, the H&E and Oil Red O staining of the liver in the normal group revealed a normal histological architecture characterized by the negligible appearance of large vacuoles accompanied by minimal lipid accumulation (Figure 4a,b). The aberrant tissue architecture with a large number of vacuoles associated with significantly higher lipid accumulation were the characteristic features of both macrovesicular and microvesicular steatosis in the liver tissues of the HFD group. These histological observations were further supported by quantitative analyses, which showed significantly higher hepatic steatosis and Oil Red O-stained areas in the HFD group vs. the normal group (Figure 4c,d). Treatment of the HFD-fed animals with all pasteurized *A. muciniphila* strains improved the histological features of the liver markedly. In addition, a significant decrease in the hepatic steatosis area was observed in the HFD group upon exposure to the three selected *A. muciniphila* strains, but not GC or BAA 835. Treatment of HFD-fed mice with GC, BAA 835, EB-AMDK 19 and EB-AMDK 27 caused a significant decrease in the hepatic Oil Red O-stained area. In particular, this parameter in the EB-AMDK 19 and EB-AMDK 27 groups was significantly lower than the GC group and the GC and BAA 835 groups, respectively.

The hepatic expressions of the vital factors of adipogenesis/lipogenesis processes were analyzed to understand the molecular mechanism for the action of *A. muciniphila* further against HFD-induced hepatic lipid accumulation and steatosis (Figure 4e–i). These results showed that the hepatic gene expression of PPARγ, SREBP1c, FAS and ACC1, as well as protein expressions of PPARγ and SREBP1c, were significantly higher in the HFD group than in the normal group. In contrast, the gene expression of these proteins and protein expressions of PPARγ and SREBP1c were downregulated significantly in the livers of the HFD-fed animals treated with all three selected pasteurized *A. muciniphila* strains. Furthermore, the hepatic expression of PPARγ and SREBP1c were significantly lower in the EB-AMDK 19 and EB-AMDK 27 groups than the GC group.

### 3.6. A. muciniphila Administration Suppressed the Adipogenesis/Lipogenesis in the Adipose Tissue of the HFD-fed Mice

**Figure 5:**
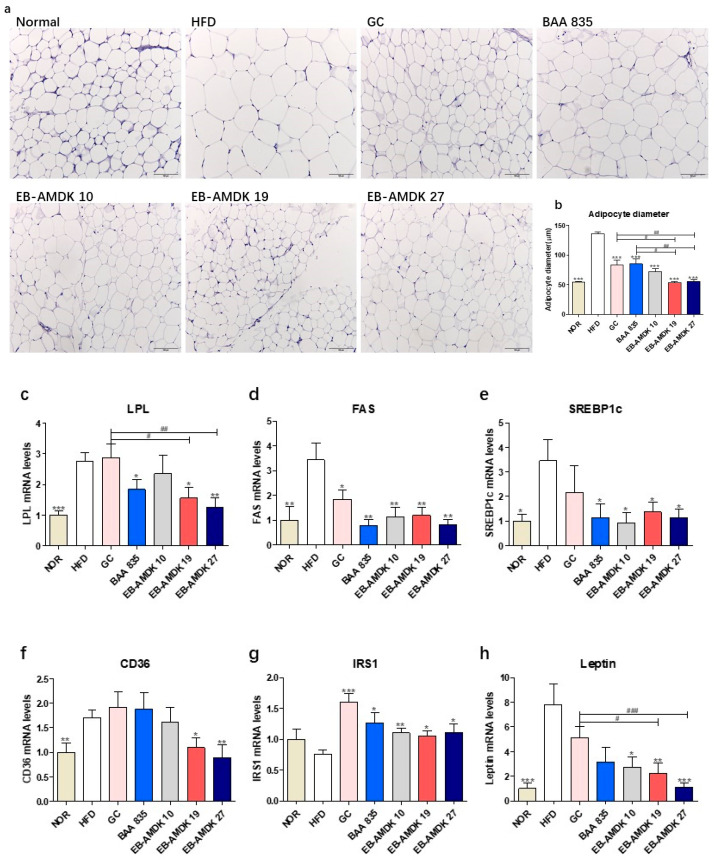
Effects of *A. muciniphila* on the adipokine profile of adipose tissue in HFD-fed mice. (**a**) Histological analysis (H&E staining) on sections of mesenteric fat tissues (*n* = 3 per condition, scale bar, 100 μm). (**b**) Average diameters of adipocytes in randomly chosen fields were measured and presented as pixels using Image-Pro Plus 6.0. (**c**) LPL, (**d**) FAS, (**e**) SREBP1c, (**f**) CD36, (**g**) IRS1 and (**h**) Leptin mRNA levels in the adipose tissues (*n* = 9–12). Data are represented as the mean ± SEM. Statistics were performed with one-way ANOVA or Student’s t-test. * *p* < 0.05, ** *p* < 0.01 and *** *p* < 0.01 versus the HFD group. ^#^ *p* < 0.05, ^##^ *p* < 0.01, ^###^ *p* < 0.001.

As anticipated, histological analysis of the mesenteric adipose tissue with H&E staining showed that compared to the normal group, the mice in the HFD group had significantly larger adipocytes, indicating the marked deposition of fat (Figure 5a,b). On the other hand, treatment of the HFD group with all pasteurized *A. muciniphila* strains reduced the average diameter of the adipocytes significantly. Furthermore, the adipocyte size was significantly smaller in both the EB-AMDK 19 and EB-AMDK 27 groups than the GC and BAA 835 groups.

Significantly higher expression of the LPL, FAS, SREBP1c and CD36 genes were observed in the adipose tissue of the HFD group vs. the normal group (Figure 5c–f). In contrast, the adipose mRNA level of FAS in the HFD-fed animals was reduced significantly upon exposure to all *A. muciniphila* strains and GC. The adipose expression of SREBP1c gene in the HFD-fed animals was also downregulated significantly upon treatment with all *A. muciniphila* strains, but not GC. On the other hand, exposure of the HFD group to EB-AMDK 19, EB-AMDK 27 and BAA 835, but not other treatments, depleted the mRNA level of adipose LPL significantly. The adipose gene expression of CD36 in the HFD-fed animals was decreased significantly by EB-AMDK 19 and EB-AMDK 27, but not the other treatments.

In addition to the above-mentioned adipogenic/lipogenic markers, this study also evaluated the impact of *A. muciniphila* strains on the adipose tissue gene expression of IRS1, a key player in the insulin-stimulated signal transduction pathway and leptin, a hormone that facilitates the regulation of feeding and energy homeostasis (Figure 5g,h). A definite, but insignificantly lower mRNA level of IRS1 was observed in the HFD group compared to the normal group. In contrast, significantly higher expression of the leptin gene was observed in the HFD group compared to the normal group. Treatment of the HFD-fed animals with the *A. muciniphila* strains increased the level of IRS1 mRNA significantly. Exposure of the HFD group to the three selected *A. muciniphila* strains, but not the other treatments, downregulated the expression of the leptin gene significantly.

### 3.7. A. muciniphila Exerted Anti-Inflammatory Effects on the Colon of the HFD-Induced Mice

**Figure 6:**
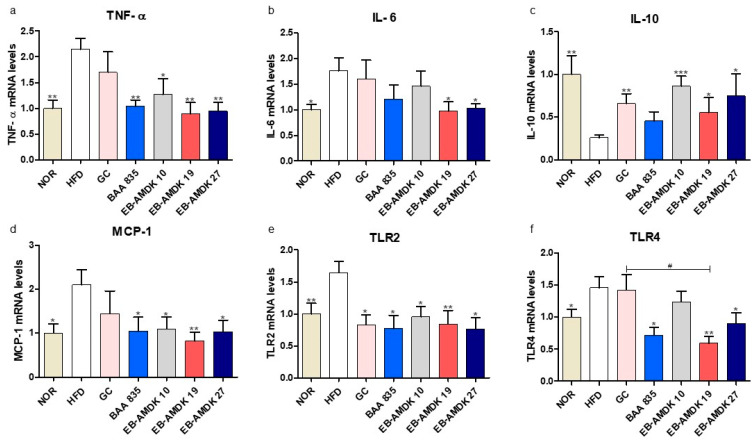
*A. muciniphila* treatment had anti-inflammatory effects on the colon of the HFD-fed mice. mRNA levels of inflammatory cytokines (**a**) TNF-α, (**b**) IL-6, (**c**) IL-10, (**d**) MCP-1, (**e**) TLR2 and (**f**) TLR4 in the intestine tissues of each group. The data are represented as the mean ± SEM. The data were analyzed with one-way ANOVA or Student’s t-test. * *p* < 0.05, ** *p* < 0.01 and *** *p* < 0.001 versus the HFD group. ^#^ *p* < 0.05.

The protective activity of pasteurized *A. muciniphila* against HFD-induced inflammation in the colon was next evaluated. This study examined the impact of *A. muciniphila* in the HFD group on the colonic gene expression of pro-inflammatory cytokines TNF-α and IL-6, anti-inflammatory cytokine IL-10, pro-inflammatory chemokine MCP-1, as well as on the toll-like receptors TLR2 and TLR4, which play vital roles in the inflammation and inflammatory signaling pathways (Figure 6a–f). These results showed that the colonic mRNA levels of TNF-α, IL-6, MCP-1, TLR2 and TLR4 were significantly higher in the HFD group than in the normal group. In contrast, the colonic expression of the IL-10 gene was significantly lower in the HFD group than in the normal group. Exposure of the HFD group to all *A. muciniphila* strains and GC depleted the mRNA level of TLR2 significantly. In contrast, the treatment of HFD-fed animals with the *A. muciniphila* strains, but not GC, downregulated the expression of TNF-α and MCP-1 genes significantly. Exposure of the HFD group to all three *A. muciniphila* strains selected and GC, but not BAA 835, increased the mRNA level of IL-10 significantly. While treatment of the HFD-fed animals with EB-AMDK 19, EB-AMDK 27 and BAA 835, but not the other treatments, decreased the expression of the TLR4 gene significantly. On the other hand, exposure of the HFD group to EB-AMDK 19 and EB-AMDK 27, but not other regimens, reduced the mRNA level of IL-6 significantly.

### 3.8. A. muciniphila Treatment Improved the Intestinal Structure and Barrier Integrity of the HFD-Fed Mice

**Figure 7:**
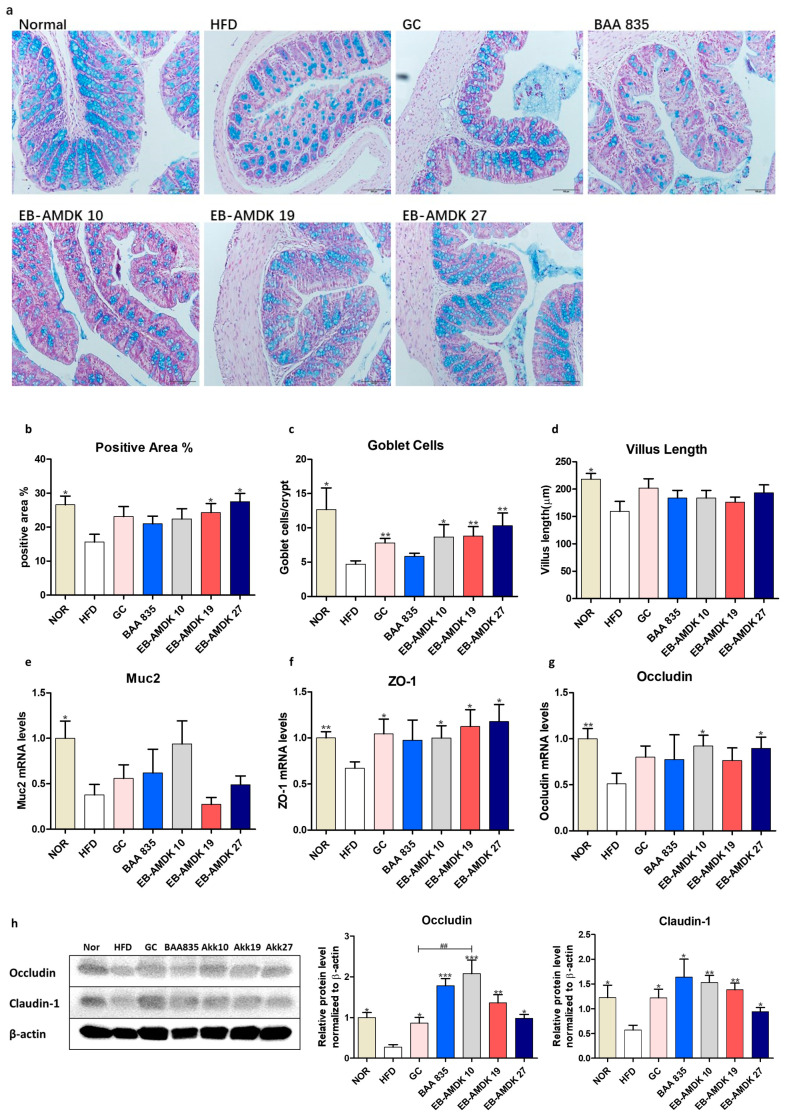
*A. muciniphila* administration improved the intestinal structure and barrier integrity of the HFD-fed mice. (**a**) Representative microscopic images demonstrating Alcian blue (AB)-staining of the colonic tissue sections of mice from different experimental groups at a magnification of 200×, (**b**) proportion of AB-positive area (%), (**c**) number of goblet cells and (**d**) length of a villus in colonic tissue sections (*n* = 3 per condition, scale bar, 100 μm). Representative mRNA expression of (**e**) Muc2, (**f**) ZO-1 and (**g**) Occludin in the intestine tissue from mice (*n* = 9–12 mice/group). (**h**) The protein expression of the intestine tissue (*n* = 4 mice/group). Data are represented as the mean ± SEM. Statistics were performed with one-way ANOVA or Student’s t-test. * *p* < 0.05, ** *p* < 0.01 and *** *p* < 0.001 versus the HFD group. ^##^ *p* < 0.01.

The histology of the AB-stained colonic tissue was analyzed to investigate the impact of pasteurized *A. muciniphila* on the intestinal structure of the HFD-fed mice (Figure 7a–d). HFD-induced obesity in the animals caused a marked disruption of in the histological architecture of the intestinal tissue. In particular, the AB-positive area, which represents both neutral and acidic mucins, was decreased significantly in response to the HFD. Moreover, the number of blue-stained mucin-secreting goblet cells and villus length in the HFD group were significantly lower than the normal group. Moreover, the gene expression of colonic mucin 2 (Muc2) was downregulated significantly in the animals fed an HFD (Figure 7e). On the other hand, a significant increase in the AB-positive area was observed in the HFD group treated with EB-AMDK 19 or EB-AMDK 27, but not the other regimens. In contrast, the number of goblet cells in the GC and all three selected *A. muciniphila* strain groups, but not the BAA 835 group, were significantly higher than the HFD group.

This study investigated the impact of pasteurized *A. muciniphila* in HFD-fed mice on the colonic gene and protein expression of vital intestinal epithelial tight junction (TJ) proteins ZO-1, occludin and claudin-1, which play a key role in maintaining the gut barrier integrity (Figure 7f–h). The levels of ZO-1 and occludin gene expression and the levels of occludin and claudin-1 protein expression were significantly lower in the mice upon fed an HFD. On the other hand, treatment of the HFD group with the GC and all three selected *A. muciniphila* strains, but not BAA 835, increased the mRNA level of ZO-1 significantly. The gene expression of occludin was upregulated significantly in the HFD group upon a treatment with EB-AMDK 10 and EB-AMDK 27, but not the other regimens. In contrast, the levels of both occludin and claudin-1 protein expression were increased significantly by all treatment regimens. Furthermore, the colonic protein level of occludin was significantly higher in the EB-AMDK 10 group than the GC group.

### 3.9. A. muciniphila Treatment Modulated the Intestinal Microbiota in the HFD-Induced Mice

**Figure 8:**
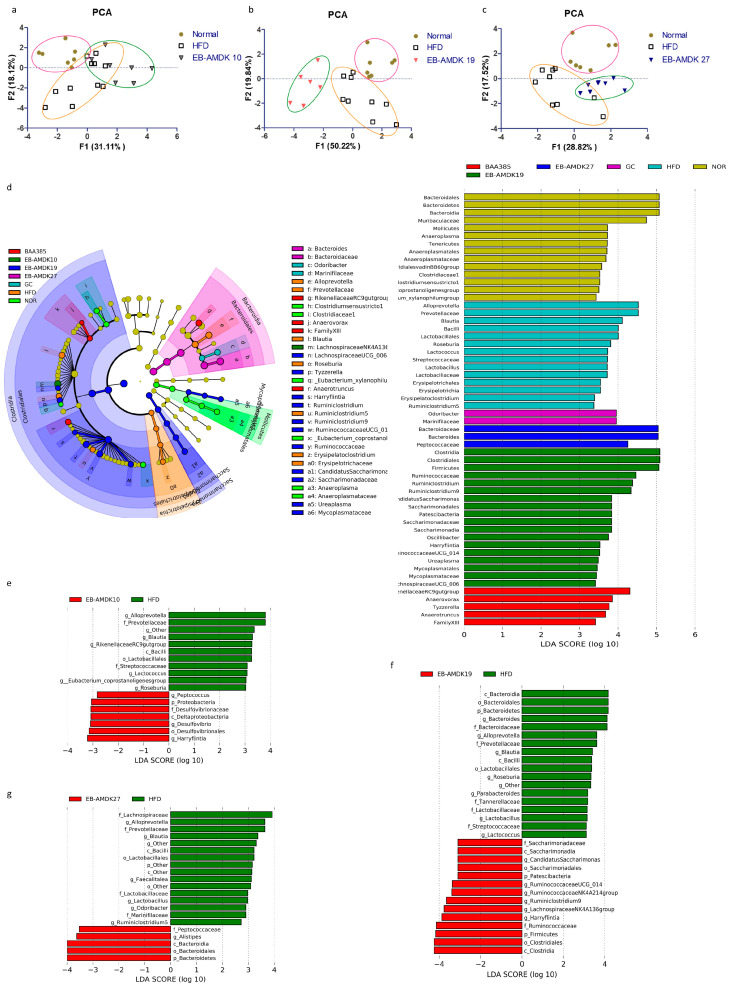
Effects of *A. muciniphila* strains on the gut microbiota composition. The gut microbiota was determined by 16S rRNA gene analysis of fecal samples from the mice. (**a**–**c**) Principal component analysis (PCA) of the gut microbiota metagenomic samples. (**d**) Cladogram generated by LEfSe analysis. The LEfSe plot shows enriched bacteria in all phenotypic categories. (**e**–**g**) LEfSe was used to identify the bacteria differentially represented between EB-AMDK 10 and HFD (**e**), EB-AMDK 19 and HFD (**f**) and EB-AMDK 27 and HFD (**g**). Only the taxa meeting a linear discriminant analysis (LDA) score threshold of two are listed.

UniFrac-based PCA of the OTUs data obtained from the fecal microbial samples showed the distinct clustering of the gut bacterial populations among the normal, HFD and each of the three selected pasteurized *A. muciniphila* strain-treated groups, but to different degrees (Figure 8a–c). In particular, distinct segregation of the gut microbial communities was observed in the EB-AMDK 19 group compared to the normal and HFD groups (Figure 8b). On the other hand, although the gut microbial population of the EB-AMDK 27 group showed segregation from that of the normal group, it exhibited partial overlap with the gut bacterial communities of the HFD group (Figure 8c). In contrast, the distribution pattern of the gut microbial population of the EB-AMDK 10 group demonstrated some overlap with that of the HFD group and remarkable segregation from the gut bacterial inhabitants of the normal group (Figure 8a).

LEfSe analysis of the 16S rRNA sequencing data showed that the relative abundance of gut microbial taxa differed among the groups (Figure 8d–g). The cladogram from the LEfSe results showed that mice fed an HFD had more abundant taxa assigned to the genus *Alloprevotella*, *Prevotellaceae, Blautia, Roseburia, Ruminiclostridium*5, *Oscillibacter, Tyzzerella,* family *Ruminococcaceae,* order *Clostridiales,* class *Clostridia,* phylum *Firmicutes* than the normal group. In contrast, the normal group exhibited more abundant genus *Lachnospiraceae* NK4A136 group, *Mollicutes, Anaeroplasmatales, Anaeroplasma, Mucoplasmataceae, Ruminococcus1,* family *Muribaculaceae, Clostridialesvadin* BB60 group, order *Bacteroidales,* class *Bacteroidia, Mollicutes* and phylum *Bacteroidetes* vs. the HFD group (Figure S4).

The genus *Peptococcus, Desulfovibrio, Harryfintia*, family *Desulfovibrionaceae* and phylum *Proteobacteria* were more abundant in the fecal samples of the EB-AMDK 10 group than in the HFD group. The EB-AMDK 19 group, however, showed a higher population of the genus *Ruminococcaceae* UCG_014, *Ruminococcaceae* NK4A214 group, *Ruminiclostridium9,* family *Ruminococcaceae,* order *Clostridiales* and class *Clostridia* than the HFD group. On the other hand, the abundance of the genus *Alistipes,* family *Peptococcaceae,* order *Bacteroidales,* class *Bacteroidia and* phylum *Bacteroidetes* were higher in the EB-AMDK 27 group compared to the HFD group. The response of the gut microbial population to the different treatments were obtained from a comparative study of the bacterial composition of the fecal samples was performed at the order and family levels among all experimental groups except for GC (Figure 8e–g). These results showed that at the order level, a significant decrease in the abundance of *Bacteriodales* and a significant increase in the population of *Clostridiales* and *Lactobacillales* occurred in the animals in response to feeding HFD.

On the other hand, treatment of the HFD group with EB-AMDK 27, but not the other *A. muciniphila* strains, increased and decreased the abundances of *Bacteriodales* and *Clostridiales* significantly, respectively (Figure 8g). In contrast, exposure of the HFD-fed animals to all *A. muciniphila* strains caused a significant decrease in the population of *Lactobacillales*. At the family level, significantly lower abundances of *Muribaculaceae* and *Rikenellaceae* and a significantly higher population of *Streptococcaceae, Prevotellaceae, Lachnospiraceae* and *Ruminococcaceae* were observed in the HFD group compared to the normal group. Treatment of the HFD-fed animals with EB-AMDK 19 and EB-AMDK 27, but not the other *A. muciniphila* strains, enhanced the population of *Rikenellaceae* significantly. Exposure of the HFD group to the *A. muciniphila* strains resulted in a significant decrease in the population of *Streptococcaceae* and *Prevotellaceae*. In contrast, treatment of the HFD-fed animals to EB-AMDK 27, but not other *A. muciniphila* strains, decreased the abundance of *Lachnospiraceae* significantly. The bacterial composition of the fecal samples in the experimental groups was compared at the genus level. These results revealed a significant decrease in the abundances of *Ruminococcaceae* NK4A214 and *Lachnospiraceae* NK4A136 groups and a significant elevation in the population of *Blautia, Lactoccus, Lactobacillus, Roseburia, Ruminiclostridium*5 and *Tyzzerella* in the mice upon fed an HFD. Treatment of the HFD-fed animals with the regimens increased the abundance of the *Ruminococcaceae* NK4A214 group significantly but reduced the population of *Blautia, Lactoccus, Lactobacillus, Roseburia and Ruminiclostridium*5 significantly. The abundance of *Roseburia* in the EB-AMDK 19 and EB-AMDK 27 groups was significantly lower than the BAA 835 group. In contrast, exposure of the HFD group to the *A. muciniphila* strains augmented the abundance of the *Lachnospiraceae* NK4A136 group significantly. On the other hand, treatment with EB-AMDK 10 and EB-AMDK 27, but not the other regimens, increased the population of *Tyzzerella* significantly (Table S4).

## 4. Discussion

Accumulating evidence supports the health-promoting effects of *A. muciniphila* [34] because it improved several metabolic parameters in overweight and obese human subjects [25]. Recently, it has been found that pasteurization of *A. muciniphila* increases its beneficial effects on metabolism [35]. This is in agreement with a clinical trial which revealed that compared to the placebo, pasteurized *A. muciniphila* significantly improved insulin sensitivity, decreased insulinemia and plasma total cholesterol in the overweight and obese subjects [25]. Adipocyte differentiation or adipogenesis is a process through which fibroblasts, such as preadipocytes, develop into mature adipocytes [36], and is associated with the onset and development of obesity [37]. The accumulation of lipid droplets, a vital index of adipogenesis, can be assessed by Oil Red O staining, which marks triglycerides and lipids in the mature adipocytes [38]. Growing evidence suggests that adipogenesis and lipogenesis are complex processes that require the involvement of several transcription factors, such as adipogenesis/lipogenesis activators, PPARγ and C/EBPα, and lipogenic enzymes, such as aP2, ACC1, LPL and FAS. PPARγ and C/EBPα, which play a key role in the regulation of adipogenesis, trigger the gene expressions of aP2, CD36, LPL and FAS to induce the synthesis of fatty acids and triglycerides [39,40]. aP2 controls the lipid and glucose metabolism in adipocytes [41]. CD36 is a scavenger receptor for a variety of ligands that regulate adipocyte proliferation and the expression of lipogenic genes during adipogenesis [40]. LPL, which plays an important role in the lipid metabolism, hydrolyzes TG in lipoprotein and facilitates the cellular uptake of chylomicron remnants and free fatty acids [42]. FAS and ACC1 catalyze the synthesis of fatty acids from acetyl-CoA and malonyl-CoA, which is an essential step in the lipogenesis process [43]. In this study, Oil Red O staining of 3T3-L1 mouse preadipocyte cells revealed the potent inhibition of cellular lipid accumulation by three *A. muciniphila* strains, EB-AMDK 10, EB-AMDK 19 and EB-AMDK 27. The anti-adipogenic/anti-lipogenic activities of these three bacterial strains were confirmed by their abilities to suppress the expression of the PPARγ, C/EBPα, aP2, CD36, ACC1, LPL and FAS genes significantly. Moreover, the level of LPL mRNA in the EB-AMDK 10 and EB-AMDK 19-treated cells and the expression of PPARγ, C/EBPα and CD36 genes in all three *A. muciniphila* strain-treated cells were significantly lower than the cells exposed to the *A. muciniphila* type strain BAA 835. This suggests that the anti-adipogenic/anti-lipogenic properties of the selected strains of *A. muciniphila* were more potent than its type strain.

The in vivo protective effects of the above-mentioned selected strains of *A. muciniphila* against obesity and metabolic dysregulation were evaluated using HFD-fed mice as a model. HFD intake induces the development of obesity, hyperinsulinemia, hyperglycemia and hypertension in animals [44]. In addition, in mice, the body weight gain, caloric intake, energy efficiency, weights of subcutaneous fat, epididymal fat, mesenteric fat and total fat, as well as the serum levels of TC and TG, were significantly higher in the HFD group than in the normal group. Previous studies showed that *A. muciniphila* exerts its beneficial effects by combating the onset and development of obesity, type 2 diabetes and other metabolic dysfunctions [21]. These results showed that all three *A. muciniphila* strains selected decreased the body weight gain, caloric intake and mesenteric and total fat weights significantly in the HFD-fed animals.

In contrast, treatment of the HFD group with EB-AMDK 19 and EB-AMDK 27, but not the other regimens, decreased the energy efficiency, subcutaneous fat weight and serum TC level significantly. Furthermore, the epididymal fat weight in the HFD group was decreased significantly by EB-AMDK 19 and EB-AMDK 27, but not BAA 835. Therefore, the EB-AMDK 19 and EB-AMDK 27 strains of *A. muciniphila* may have a more potent beneficial impact on the obesity-related parameters in the HFD-fed animals than in the type strain and GC, which is in agreement with our in vitro results.

Several lines of evidence suggest that insulin resistance is associated with obesity, especially visceral adipose tissue accumulation [45]. In agreement with the above, the fasting serum glucose concentration, peak OGTT glucose level, OGTT AUC, serum insulin concentration and HOMA-IR, which are the parameters for measuring insulin resistance, glucose tolerance and homeostasis, were significantly higher in the HFD-induced obese group than in the normal group. Prolonged hyperlipidemia may contribute to the increased production of glucose via the increased expression of G6Pase, a multicomponent enzyme predominantly expressed in the liver [46]. This enzyme, which is vital for endogenous glucose production, catalyzes the hydrolysis of glucose-6-phosphate to glucose in the terminal step of glycogenolysis and gluconeogenesis processes [47]. Moreover, the gene expression of hepatic G6Pase was significantly higher in the HFD group than in the normal group. In addition, a significant downregulation of the colonic gene expression of the gut hormone peptides GLP-1 and PYY was observed in the animals in response to feeding HFD. GLP-1, which is used widely to treat diabetes and obesity, promotes glucose-induced insulin secretion, inhibits glucagon secretion and suppresses the appetite and food intake by slowing gastric emptying and increasing satiety [48]. On the other hand, PYY, which can modulate the body weight by inhibiting the appetite and increasing energy expenditure [49], has been shown to reduce excess adiposity and improve glucose tolerance [50].

Accumulating evidence suggests that reduced fat accumulation in the liver and skeletal muscle may increase the binding activity of the insulin receptor to insulin on the cell membrane and improve insulin sensitivity and glucose homeostasis [51]. In keeping with this, the treatment of HFD-fed animals with the three selected *A. muciniphila* strains that showed a marked anti-obesity effect, decreased the peak OGTT glucose level, OGTT AUC, serum insulin level, HOMA-IR significantly, as well as hepatic gene expression of hepatic G6Pase and upregulated the colonic gene expression of PYY significantly. In contrast, exposure of the HFD group to EB-AMDK 10 and EB-AMDK 27, but not to the other treatments, increased the colonic mRNA level of GLP-1 significantly. Furthermore, OGTT AUC was significantly lower in the EB-AMDK 19 group than the GC group, whereas this parameter in the EB-AMDK 27 group was significantly lower than in the GC and BAA 835 groups. In contrast, exposure of the HFD group to EB-AMDK 19 and EB-AMDK 27, but not the other treatments, decreased the serum fasting glucose level significantly. Furthermore, treatment of the HFD-fed mice to EB-AMDK 19 and EB-AMDK 27 upregulated the hepatic gene expression of GLUT2 significantly. GLUT2 is a facilitative glucose transporter that promotes the bidirectional transfer of glucose across the liver cell membrane [52]. Overall, in the HFD-fed animals, EB-AMDK 19 and EB-AMDK 27 strains of *A. muciniphila* exert a more potent beneficial impact on insulin resistance and glucose homeostasis compared to its type strain and GC.

Several lines of evidence indicate that in animals, HFD intake induces obesity accompanied by liver damage, similar to the phenotype evident in humans suffering from NAFLD [44]. In the present study, in parallel with the increase in body weight, the liver weight of the mice was enlarged significantly upon the consumption of the HFD. The deposition of lipids in the liver and insulin resistance are considered the key factors contributing to hepatic steatosis development [53]. The present histological studies and Oil Red O staining of liver tissue sections from the HFD-fed animals revealed the typical characteristics of hepatic steatosis in association with markedly higher lipid accumulation. This was associated with liver damage because the serum level of GPT, a vital marker of liver injury, was increased significantly in the animals in response to HFD consumption. In addition, the hepatic gene expression of PPARγ, FAS, ACC1 and SREBP1c, the key factors regulating adipogenesis/lipogenesis processes, were significantly higher in the HFD group than in the normal group. Accumulating evidence suggests that SREBPs directly trigger the expression of more than 30 genes dedicated to the synthesis and uptake of cholesterol, fatty acids, triglycerides and phospholipids, as well as the NADPH cofactor required for the synthesis of these molecules [54]. In particular, treatment of HFD-fed animals with all three selected *A. muciniphila* strains reduced the weight and steatosis area of the liver and serum level of GPT significantly, improved the histological architecture of the liver markedly and suppressed the hepatic gene expression of PPARγ, SREBP1c, FAS, ACC1 and hepatic protein expressions of PPARγ and SREBP1c significantly. Furthermore, exposure of the HFD group to EB-AMDK 10 and EB-AMDK 19 decreased the levels of hepatic lipid accumulation and the serum level of GOT significantly, which is another key marker of liver injury. These results corroborate the in vivo hepatoprotective activity of the selected strains of *A. muciniphila* and their anti-adipogenic/anti-lipogenic on the liver of the HFD-fed animals.

Consistent with the higher mass, as described previously, mesenteric adipose tissue also increased in size with larger adipocytes in the HFD group than the normal group. Adipocyte hypertrophy was found to be the major mechanism for the expansion of adipose tissue [55]. In the present study, exposure of the HFD group by the *A. muciniphila* strains decreased the average diameter of the adipocytes significantly. In addition, significantly higher expression of the LPL, FAS, SREBP1c, CD36 and leptin genes was observed in the adipose tissue of the HFD group than in the normal group. Leptin, a hormone secreted from fat cells, is an appetite suppressor and key regulator of the adipose tissue mass and body weight. Overexpression of the leptin gene has been observed in the subcutaneous adipose tissue of morbidly obese individuals [56]. The induction of obesity by prolonged exposure to an HFD also caused a rise in the leptin level (hyperleptinemia). In contrast, the sensitivity of tissues to leptin was diminished, leading to insulin resistance [57]. Furthermore, the adipose tissue level of IRS1 mRNA was lower in the HFD group than in the normal group but the difference was not significant. Reduced adipose tissue IRS-1 expression was reported in obese subjects [58], which impairs the downstream insulin signaling and the development of insulin resistance accompanied by increased adiposity [59]. On the other hand, the adipose levels of FAS, SREBP1c and leptin gene expression were downregulated significantly, and the adipose level of IRS1 mRNA was elevated significantly in the HFD-fed animals exposed to all three selected *A. muciniphila* strains. On the other hand, treatments of the HFD group with EB-AMDK 10 and EB-AMDK 27 downregulated the adipose gene expression of LPL and CD36 significantly. Reversal of the expression of the abovementioned leptin gene in the HFD-fed animals by *A. muciniphila* supplementation might improve the leptin sensitivity and suppress calorie intake and energy efficiency, as observed in the present study.

While an overweight status and obesity are generally considered to be the outcome of an imbalance in energy homeostasis, accumulating evidence suggests that metabolic disorder is associated with low-grade chronic inflammation [60]. An HFD elevates the LPS level in serum, causing endotoxemia [61]. Furthermore, emerging evidence suggests that HFD could induce the production of pro-inflammatory cytokines, including IL-1, IL-6, TNF-α, pro-inflammatory chemokine MCP-1 and toll-like receptors TLR2 and TLR4, in a variety of tissues, including the colon. These eventually trigger low-grade inflammation that may be associated with obesity, insulin resistance and other metabolic disorders [61,62,63]. In agreement with this, the colonic gene expression of TNF-α, IL-6 and MCP-1, and TLR2 and TLR4 were significantly higher in the HFD group than the normal group. TLR2 and TLR4 contribute to metabolic syndrome associated with HFD-induced obesity, insulin resistance and tissue inflammation [64]. In addition, the colonic mRNA level of IL-10, a potent anti-inflammatory cytokine that plays a crucial role in preventing inflammatory and autoimmune pathologies [65], was significantly lower in the HFD group than in the normal group. Previous studies reported that *A. muciniphila* could elevate the levels of anti-inflammatory cytokines [66] and reduce the pro-inflammatory conditions to improve the systemic inflammation caused by HFD in mice [67]. Consistent with these findings, these results showed that treatment of the HFD group with all three *A. muciniphila* strains selected increased the colonic gene expression of IL-10 significantly and reduced the colonic mRNA levels of TNF-α, MCP-1 and TLR2. On the other hand, exposure of the HFD-fed animals to EB-AMDK 19 and EB-AMDK 27 downregulated the expression of IL-6 and TLR4 genes in the colon significantly. In contrast, exposure of HFD-fed animals to BAA 835 did not have any significant effect on the gene expression of IL-6 and IL-10.

Because of the above-mentioned anti-inflammatory properties of the selected *A. muciniphila* strains, this study next evaluated the protective activity of these bacterial strains against an HFD-induced insult on the intestinal structure and the factors related to gut permeability. The anti-inflammatory process is associated with a lowering of the intestinal permeability in diet-induced obesity mice by improving the gut barrier integrity [68,69]. In this study, an abrupt histological architecture was observed in the AB-stained intestinal tissue of the animals fed an HFD. In particular, the AB-positive area representing both neutral and acidic mucins was reduced significantly after feeding them an HFD. The number of blue-stained goblet cells and villus length in the HFD group were significantly lower than the normal group. This concurs with previous studies, where an HFD was accompanied by a distorted microvilli structure and disoriented crypts [70] as well as a significantly lower number of goblet cells [71]. In addition, the colonic gene expression of Muc2 was significantly suppressed in the animals after being fed an HFD. In contrast, a significant increase in the AB-positive area was observed in the HFD-fed animals in response to a treatment with both EB-AMDK 19 and EB-AMDK 27, but not the other regimens. Furthermore, the goblet cell counts in the GC and the three selected *A. muciniphila* strain groups were significantly higher than the HFD group but not the BAA 835 group. Goblet cells are specialized epithelial cells that protect the intestine by synthesizing and secreting several mediators, including the Muc2 [72], the main component of the mucus layer and the small peptide, trefoil factor 3 (TFF3) [73]. Exposure to *A. muciniphila* augmented the number of goblet cells and mucus secretion, restoring the thickness of the intestinal mucus damaged by the HFD, thereby recovering the epithelial barrier integrity [22].

Accumulating evidence suggests that the epithelial tight junction proteins, including ZOs, occludin and claudins, are the key players in regulating the intestinal-barrier function and modulating the intestinal permeability [74]. An HFD impairs the gut barrier function, an event that is mediated via a mechanism associated with the reduced intestinal expressions of ZO-1, occludin and claudin-1 [11,68]. These are in keeping with the present results showing that the colonic gene expression of ZO-1 and occludin and the protein expression of occludin and claudin-1 were suppressed in the animals fed an HFD. On the other hand, the treatment of the HFD group with the three selected *A. muciniphila* strains increased the gene expression of ZO-1 and the protein expression of occludin and claudin-1 significantly. In contrast, the gene expression of occludin was upregulated significantly in the HFD group upon a treatment with EB-AMDK 10 and EB-AMDK 27. In contrast, exposure of the HFD-fed animals to BAA 835 did not have any significant effect on the gene expressions of ZO-1 and occludin.

Several lines of evidence indicate an association between obesity and gut microbiota, which plays significant roles in maintaining the nutrient and energy balance, lipid metabolism and immune response of the host [75]. The diet is considered one of the vital factors that determine the gut microbial composition [76]. Feeding animals an HFD alters the gut microbial diversity, which correlates with the obesity-associated metabolic parameters [13,63]. The effects of the gut microbiota on the host varies according to their composition and population [77]. Therefore, diversity is a critical factor in predicting the potential roles of the gut microbiota. The current line of evidence indicates that a treatment with probiotics could ameliorate intestinal dysbiosis and obesity by modulating the gut microbiota [78]. The phylogenetic diversity of the fecal bacterial community was examined by 16S ribosomal RNA gene sequence analysis to determine how *A. muciniphila* supplementation could affect the gut microbial population in the HFD-fed mice. The UniFrac-based PCA demonstrated distinct clustering of the gut microbial populations among the normal, HFD and each of the three *A. muciniphila* strain-treated groups, but to different degrees, indicating the treatment-specific response of the gut bacterial distribution.

Further analysis of the 16S rRNA sequencing data showed that the relative abundance of gut microbial taxa differed among the experimental groups. At the genus level, a significant decrease in the abundance of *Ruminococcaceae NK4A214* and *Lachnospiraceae NK4A136* groups and a significant elevation in the population of *Blautia, Lactoccus, Lactobacillus, Roseburia, Ruminiclostridium*5 and *Tyzzerella* were observed in the mice fed an HFD. Accumulating evidence suggests that HFD-induced modulation in the gut microbial community is associated with an enhanced intestinal permeability due to the disintegration of the gut barrier, which eventually leads to the development of metabolic endotoxemia, inflammation and metabolic disorders [13,63]. These findings are also in agreement with previous reports showing negative correlations of the genera *Ruminococcaceae NK4A214* and *Lachnospiraceae NK4A136* groups with HFD consumption [79,80]. One of the vital functions of *Ruminococcaceae* and *Lachnospiraceae* produced short-chain fatty acids (SCFA), such as butyric acid and acetic acid [81,82]. SCFA plays an important role in maintaining a healthy condition because they may minimize the risk of many diseases, including type 2 diabetes and obesity [83]. In a recent study, however, *Blautia, Lactoccus and Tyzzerella* showed significant negative correlations with SCFAs [84]. Furthermore, the genus *Roseburia* may promote weight gain [85]. In contract, *Ruminiclostridium* was positively correlated with the obesity-related parameters, including the lipid profile and the hepatic lipogenesis [86]. In the present study, all three selected strains of *A. muciniphila* affected the gut microbial communities of the HFD group at the genus level by increasing the abundance of *Ruminococcaceae NK4A214* and *Lachnospiraceae NK4A136 groups* while decreasing the population of *Blautia, Lactoccus, Lactobacillus, Roseburia* and *Ruminiclostridium5*. In contrast, the abundance of *Tyzzerella* in the HFD group decreased significantly after exposure to EB-AMDK 10 and EB-AMDK 27. The population of *Bacteriodales, Bacteroidaceae* and *Bacteroides* in the HFD-fed animals was increased significantly by exposure to EB-AMDK 27. In a previous clinical study, *Bacteriodales* had a significant negative correlation with the body weight and body mass index, whereas *Bacteroides* showed a significant negative correlation with the waist circumference of the subjects [87].

## 5. Conclusions

The screening approach involving in vitro and in vivo studies led to the identification of three new *A. muciniphila* strains- EB-AMDK 10, EB-AMDK 19 and EB-AMDK 27, which prevented HFD-induced obesity and related metabolic disorders. To the best of the authors’ knowledge, this is the first report showing that supplementation with the above three *A. muciniphila* strains suppressed body weight gain, calorie intake, fat mass, adipogenesis/lipogenesis, serum TC level and inflammatory insult. In addition, it restored the damaged gut structure and integrity and improved the hepatic functions, insulin resistance and glucose homeostasis in the HFD-fed mice. These effects were mediated by several mechanisms involving the induction/suppression of the expressions of several genes and proteins. For many of the parameters, the three strains selected had a more potent beneficial impact compared to the type strain BAA835 and GC. Furthermore, the HFD and HFD + *A. muciniphila* groups showed a different distribution pattern of the gut microbial population. This suggests that modulation of the gut microbial population with the selected *A. muciniphila* strains, at least in part, might have a beneficial impact against metabolic disorders.

## Figures and Tables

**Table 1:** Impact of three selected *A. muciniphila* strains on the expression of the adipogenic and lipogenic genes in 3T3-1 adipocytes.

Parameters	Control	BAA 835	EB-AMDK 10	EB-AMDK 19	EB-AMDK 27
**adipogenic/lipogenic markers**					
PPARγ	100.00 ± 2.03	62.77 ± 0.78 ***	41.80 ± 2.29 ***^,a^	39.48 ± 2.52 ***^,a^	47.07 ± 2.76 ***^,a^
C/EBPα	100.00 ± 5.99	62.20 ± 1.07 ***	47.39 ± 4.48 ***^,a^	15.55 ± 1.19 ***^,a^	19.76 ± 4.15 ***^,a^
CD36	100.00 ± 4.59	42.62 ± 0.56 ***	27.06 ± 0.31 ***^,a^	23.67 ± 3.52 ***^,a^	29.15 ± 2.00 ***^,a^
**lipogenic enzymes**					
aP2	100.00 ± 2.25	53.59 ± 1.17 ***	41.84 ± 1.07 ***	41.80 ± 7.35 ***	50.30 ± 3.76 ***
ACC1	100.00 ± 14.81	37.99 ± 2.97 ***	19.79 ± 6.31 ***	17.95 ± 4.12 ***	25.84 ± 8.47 ***
LPL	100.00 ± 3.82	44.23 ± 5.52 ***	22.59 ± 6.38 ***^,a^	18.57 ± 2.02 ***^,a^	27.97 ± 2.63 ***
FAS	100.00 ± 7.55	27.28 ± 4.50 ***	16.15 ± 9.12 ***	11.36 ± 3.98 ***	25.07 ± 4.08 ***

The values are presented as the mean ± SEM. *** *p* < 0.001 versus the control group. ^a^ *p* < 0.05 versus the BAA 835 group.

## Supplementary Materials

The following are available online at https://www.mdpi.com/2076-2607/8/9/1413/s1, Figure S1: Genetic diversity of *A. muciniphila* strains. Figure S2: *A. muciniphila* strains improved glucose homeostasis in HFD-induced obese mice. Figure S3: *A. muciniphila* strains mitigate liver injury. Figure S4: *A. muciniphila* treatment modulated the composition of the gut microbiota. Table S1: Information on the fecal sample from healthy volunteers. Table S2: Primer sequences for PCR. Table S3: Effect of *A. muciniphila* on adiposity, liver weight, and energy efficiency in mice. Table S4: Modulation of gut microbiota composition by the treatment of *A. muciniphila*.
