# Supplementary material for: Beneficial Effects of Newly Isolated Akkermansia muciniphila Strains from the Human Gut on Obesity and Metabolic Dysregulation
Source: Microorganisms. 2020 Sep 14;8(9):1413. doi: 10.3390/microorganisms8091413 (PMC7564497; doi:10.3390/microorganisms8091413)
Supplement: Supplementary file 1 [file microorganisms-08-01413-s001.pdf]

microorganisms

Supplementary Figure 1: Genetic diversity of *A. muciniphila* strains. (a) phylogenetic tree of 16S rRNA gene, and (b) phylogenetic tree of MLST genes.

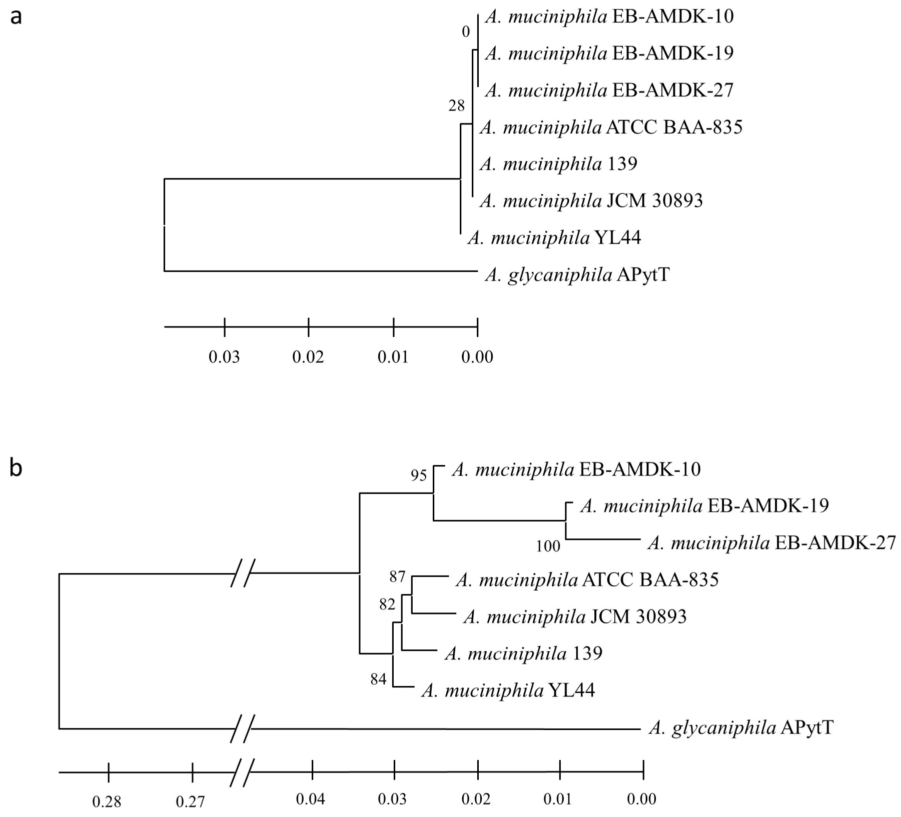

Supplementary Figure 2: *A. muciniphila* strains improved glucose homeostasis in HFD-induced obese mice. (a) Fasting glucose at 0min of OGTT. (b) 30 min glucose level. (c) Areas under the curve of OGTT measured between 0 and 120 min after glucose administration. The data are represented as the mean ± SEM (n = 9-12). Statistical analysis was performed with one-way ANOVA or Student's t-test. **p* < 0.05, ***p* < 0.01 and ****p* < 0.001 versus the HFD group. #*p* < 0.05, ##*p* < 0.01.

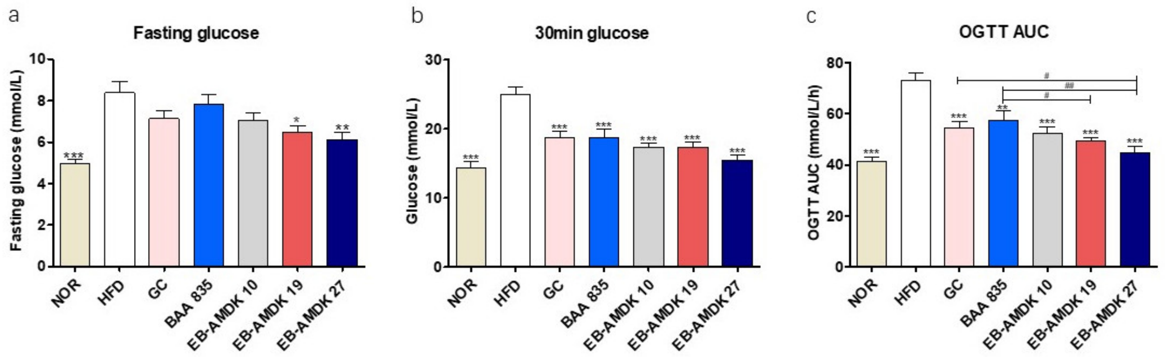

Supplementary Figure 3: *A. muciniphila* strains mitigate liver injury. (a) Glutamic oxaloacetic transaminase (GOT); (b) Glutamic pyruvic transaminase (GPT) were measured in the serum of each mouse. The data are represented as the mean ± SEM (n = 9-12). Statistics were performed with one-way ANOVA or Student's t-test. **p* < 0.05, ***p* < 0.01 and ****p* < 0.001 versus the HFD group. $^{\#}p < 0.05$, $^{\#\#}p < 0.01$.

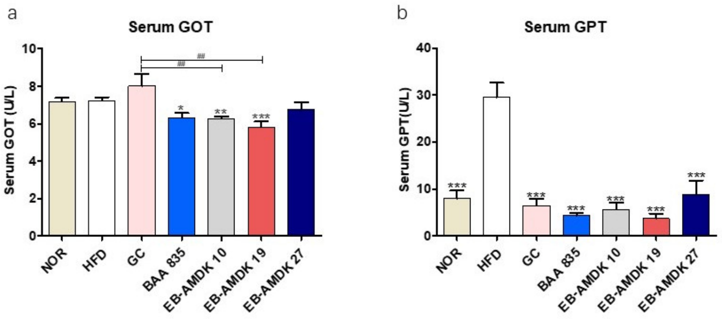

Supplementary Figure 4: *A. muciniphila* treatment modulated the composition of the gut microbiota. LEfSe was used to identify the bacteria represented differentially between the normal group and the HFD group.

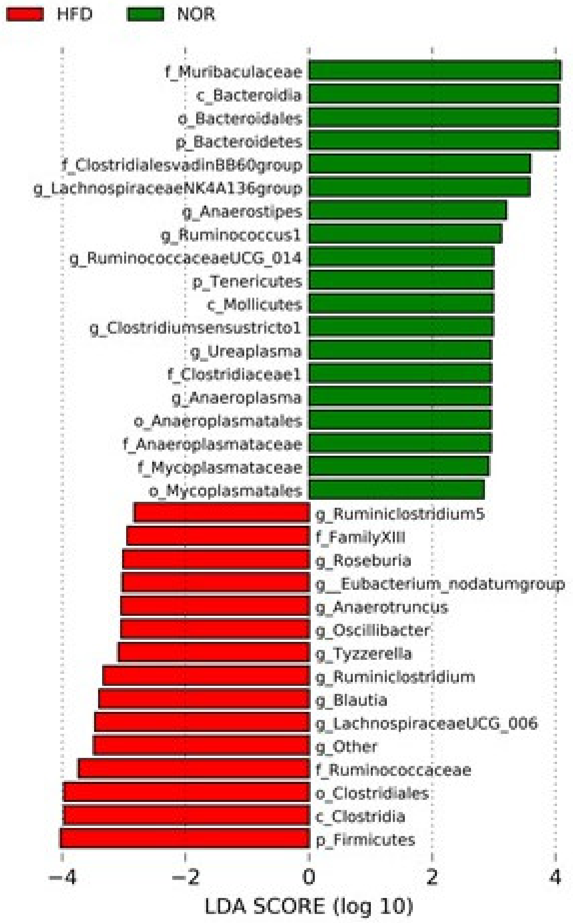

Supplementary table 1: Information on the fecal sample from healthy volunteers.

Group	Sample	Unique identification number	Gender	Age	Height (cm)	Body weight (kg)	BMI (kg/m²)
**Group01**	AMDK-7	CGT039	F	38	159	54	21.4
**Group02**	AMDK-29	CGT078	F	38	165	52	19.1
	AMDK-30	CGT078	F	38	165	52	19.1
**Group03**	AMDK-3	CGT002	F	4	95	12	13.3
	AMDK-4	CGT003	F	61	152	50	21.6
**Group04**	AMDK-23	CGT068	M	51	173	61	20.4
	AMDK-24	CGT068	M	51	173	61	20.4
	AMDK-25	CGT068	M	51	173	61	20.4
	AMDK-26	CGT068	M	51	173	61	20.4
**Group05**	AMDK-46	CGT092	F	30	157	58	23.5
	AMDK-47	CGT092	F	30	157	58	23.5
	AMDK-48	CGT092	F	30	157	58	23.5
	AMDK-49	CGT092	F	30	157	58	23.5
**Group06**	AMDK-1	CGT001	M	35	175	72	23.5
**Group07**	AMDK-15	CGT060	M	3	95	12.5	13.9
	AMDK-16	CGT060	M	3	95	12.5	13.9
	AMDK-17	CGT060	M	3	95	12.5	13.9
	AMDK-18	CGT060	M	3	95	12.5	13.9
**Group08**	AMDK-8	CGT031	M	44	173	71	23.7
**Group09**	AMDK-27	CGT074	F	45	150	52	23.1
	AMDK-28	CGT074	F	45	150	52	23.1
**Group10**	AMDK-2	CGT001	M	35	175	72	23.5
	AMDK-6	CGT005	F	37	157	46	18.7
	AMDK-10	CGT007	M	7	120	25	17.4
	AMDK-11	CGT007	M	7	120	25	17.4
	AMDK-12	CGT007	M	7	120	25	17.4
	AMDK-13	CGT007	M	7	120	25	17.4
	AMDK-14	CGT007	M	7	120	25	17.4
**Group11**	AMDK-5	CGT006	M	63	173	67	22.4
	AMDK-19	CGT034	F	35	167	65	23.3
	AMDK-20	CGT034	F	35	167	65	23.3
	AMDK-21	CGT034	F	35	167	65	23.3
	AMDK-22	CGT034	F	35	167	65	23.3
	AMDK-33	CGT079	F	60	157	54	21.9
	AMDK-34	CGT079	F	60	157	54	21.9
	AMDK-35	CGT079	F	60	157	54	21.9
	AMDK-36	CGT079	F	60	157	54	21.9
**Group12**	AMDK-31	CGT076	F	40	159	62	24.5
	AMDK-37	CGT071	M	14	151	47	20.6

	AMDK-38	CGT071	M	14	151	47	20.6
	AMDK-39	CGT085	F	7	112	25	19.9
**Group13**	AMDK-40	CGT085	F	7	112	25	19.9
	AMDK-41	CGT085	F	7	112	25	19.9

Supplementary table 2: Primer sequences for PCR.

Gene		Primer Sequence (5'–3')
PPARγ	F:	GCCCTTTGGTGACTTTATGGA
	R:	GCAGCAGGTTGTCTTGGATG
C/EBPα	F:	AGCAACGAGTACCGGGTACG
	R:	TGTTTGGCTTTATCTCGGCTC
Ap2	F:	AGTGAAAACTTCGATGATTACATGAA
	R:	GCCTGCCACTTTCCTTGTG
CD36	F:	TTGTACCTATACTGTGGCTAAATGAGA
	R:	CTTGTGTTTTGAACATTTCTGCTT
ACC1	F:	CCTCCGTCAGCTCAGATACA
	R:	TTTACTAGGTGCAAGCCAGACA
LDLR	F:	GAACTCAGGGCCTCTGTCTG
	R:	CAGGCTGGATGTCTCTGTGA
LPL	F:	TTCCAGCCAGGATGCAACA
	R:	GGTCCACGTCTCCGAGTCC
FAS	F:	AGGGGTCGACCTGGTCCTCA
	R:	GCCATGCCCAGAGGGTGGTT
SREBP1c	F:	GGAGCCATGGATTGCACATT
	R:	GGCCCGGGAAGTCACTGT
GLUT2	F:	GGCTAATTTCAGGACTGGTT
	R:	TTTCTTTGCCCTGACTTCCT
G6Pase	F:	GACTCCCAGGACTGGTTCAT
	R:	GGGCGTTGTCCAAACAGAAT
IRS1	F:	TTTGAAGACCATAACCCACCA
	R:	ATTACACCAGTTCGTCCCTTTC
Leptin	F:	GCCAGGCTGCCAGAATTG
	R:	CTGCCCCCCAGTTTGATG
TNFα	F:	GACCCTCACACTCAGATCATCTTCT
	R:	CCACTTGGTGGTTTGCTACGA
IL-6	F:	CCTCTGGTCTTCTGGAGTACC
	R:	ACTCCTTCTGTGACTCCAGC
IL-10	F:	ATAACTGCACCCACTTCCCA
	R:	GGGCATCACTTCTACCAGGT
IL-1β	F:	TGCCACCTTTTGACAGTGATG
	R:	AAGGTCCACGGGAAAGACAC
MCP-1	F:	AAGAGATCAGGGAGTTTGCT
	R:	CTGCCTCCATCAACCACTTT
TLR2	F:	AAGGAGGTGCGGACTGTTTC
	R:	GAGCCAAAGAGCTCGTAGC
TLR4	F:	CCTGATGACATTCCTTCTTCAAC
	R:	TTGTTTCAATTTCACACCTGGATAAA
GLP-1	F:	GGCACATTCACCAGCGACTAC

	R:	CAATGGCGACTTCTTCTGGG
PYY	F:	CGGCAGCGGTATGGAAAAA
	R:	TGTGAAGAGCAGTTTGGAGAACA
Lyz1	F:	GCCAAGGTCTACAATCGTTGTGAGTTG
	R:	CAGTCAGCCAGCTTGACACCACG
Muc2	F:	CCATTGAGTTTGGGAACATGC
	R:	TTCGGCTCGGTGTTCAGAG
ZO-1	F:	TTTTTGACAGGGGGAGTGG
	R:	TGCTGCAGAGGTCAAAGTTCAAG
Occludin	F:	ATGTCCGGCCGATGCTCTC
	R:	TTTGGCTGCTCTTGGGTCTGTAT

Supplementary table 3: Effect of *A. muciniphila* on adiposity, liver weight, and energy efficiency in mice.

Parameters	NOR	HFD	GC	BAA 835	EB-AMDK 10	EB-AMDK 19	EB-AMDK 27
Energy efficiency (g/kcal/mouse)	0.0113±0.0012**	0.0206±0.0010	0.0171±0.0012	0.0170±0.0015	0.0173±0.0020	0.0144±0.0023*	0.0155±0.0014*
Liver weight (g)	1.23±0.07***	1.77±0.08	1.07±0.08***	1.16±0.09***,b	1.09±0.08***	1.03±0.04***	0.94±0.04***
Subcutaneous fat (g)	0.47±0.03***	1.56±0.05	1.30±0.04	1.35±0.06	1.44±0.09	0.97±0.11***,a,b	1.09±0.09***,a,b
Epididymal fat (g)	1.47±0.07***	2.83±0.10	2.25±0.13**	2.36±0.25	2.40±0.21	2.00±0.19***	2.27±0.18*
Mesenteric fat (g)	0.48±0.03***	1.78±0.12	1.10±0.18**	0.93±0.12***	1.16±0.17*	0.93±0.14***	0.82±0.11***

The data are represented as the mean ± SEM (n=9-12). Statistics were performed with one-way ANOVA or Student's t-test. *$p < 0.05$, **$p < 0.01$ and ***$p < 0.001$ versus the HFD group. $^{a}p < 0.05$ versus the BAA 835 group. $^{b}p < 0.05$ versus the GC group.

Supplementary table 4: Modulation of gut microbiota composition by the treatment of *A. muciniphila.*

Parameters	NOR	HFD	BAA 835	EB-AMDK 10	EB-AMDK 19	EB-AMDK 27
**Order**						
Bacterioidales	0.667±0.031*	0.540±0.024	0.613±0.033	0.544±0.056	0.478±0.057	0.686±0.018*
Clostridiales	0.284±0.030*	0.431±0.022	0.359±0.034	0.401±0.054	0.454±0.052	0.299±0.018*
Lactobacillales	0.016±0.004*	0.029±0.006	0.014±0.003*	0.011±0.003**	0.007±0.001**	0.011±0.002**
**Family**						
Bacteroidaceae	0.300±0.027	0.259±0.027	0.307±0.035	0.285±0.046	0.190±0.028	0.411±0.026*,a
Muribaculaceae	0.229±0.022***	0.104±0.016	0.162±0.025	0.169±0.012	0.149±0.031	0.151±0.012
Rikenellaceae	0.116±0.019*	0.045±0.011	0.094±0.021	0.082±0.010	0.112±0.023*	0.113±0.015*
Streptococcaceae	0.006±0.002*	0.016±0.003	0.009±0.002*	0.005±0.001**	0.005±0.001**	0.008±0.001*
Prevotellaceae	0.027±0.007*	0.078±0.022	0.034±0.009*	0.002±0.001***	0.019±0.008*	0.024±0.012*
Lachnospiraceae	0.157±0.015*	0.242±0.021	0.171±0.025	0.239±0.029	0.213±0.033	0.124±0.010**
Ruminococcaceae	0.089±0.013**	0.177±0.011	0.156±0.015	0.180±0.025	0.221±0.028	0.124±0.010
**Genus**						
Bacteroides	0.300±0.027	0.259±0.027	0.293±0.033	0.268±0.043	0.178±0.027	0.404±0.024*
Ruminococcaceae NK4A214 group	0.0010±0.0002*	0.0004±0.0002	0.0011±0.0002*	0.0010±0.0001**	0.0015±0.0003**	0.0013±0.0003*
Lachnospiraceae NK4A136 group	0.075±0.008***	0.024±0.006	0.081±0.028*	0.100±0.028**	0.105±0.016***	0.046±0.008*
Blautia	0.003±0.001***	0.027±0.005	0.011±0.003**	0.010±0.003**	0.006±0.003**	0.006±0.002***
Lactococcus	0.008±0.003*	0.016±0.003	0.009±0.002*	0.005±0.001**	0.005±0.001**	0.008±0.002*
Lactobacillus	0.005±0.002*	0.014±0.004	0.005±0.002*	0.007±0.003*	0.003±0.001*	0.004±0.001*
Roseburia	0.007±0.002**	0.016±0.003	0.010±0.002*	0.005±0.002**	0.004±0.001***,a	0.003±0.001***,a
Ruminiclostridium 5	0.0011±0.0004**	0.0041±0.0009	0.0019±0.0003**	0.0017±0.0005**	0.0023±0.0004*	0.0015±0.0002**
Tyzzerella	0.0006±0.0003***	0.0111±0.0016	0.0077±0.0024	0.0052±0.0009*	0.0077±0.0015	0.0045±0.0009*

The values are presented as the mean ± SEM. Statistics were performed with one-way ANOVA or Student's t-test. *$p < 0.05$, **$p < 0.01$ and ***$p < 0.001$ versus the HFD group. $^{a}p < 0.05$ versus the BAA 835 group.

© 2020 by the author. Licensee MDPI, Basel, Switzerland. This article is an open access article distributed under the terms and conditions of the Creative Commons Attribution (CC BY) license (http://creativecommons.org/licenses/by/4.0/).
